# Proteomic profiling of human intraschisis cavity fluid

**DOI:** 10.1186/s12014-017-9148-y

**Published:** 2017-04-24

**Authors:** Dhandayuthapani Sudha, Mahdokht Kohansal-Nodehi, Purnima Kovuri, Srikanth Srinivas Manda, Srividya Neriyanuri, Lingam Gopal, Pramod Bhende, Subbulakshmi Chidambaram, Jayamuruga Pandian Arunachalam

**Affiliations:** 1 SN ONGC Department of Genetics and Molecular Biology, Vision Research Foundation, Chennai, India; 2 School of Biotechnology, SASTRA University, Thanjavur, India; 3 Department of Neurobiology, Max Planck Institute for Biophysical Chemistry, Göttingen, Germany; 4 Department of Biochemistry and Molecular Biology, Pondicherry University, Puducherry, India; 5 Institute of Bioinformatics, Bengaluru, India; 6 Elite School of Optometry, Unit of Medical Research Foundation, Chennai, India; 7 Shri Bhagwan Mahavir Vitreo-Retinal Services, Medical Research Foundation, Chennai, India; 8 Central Inter-Disciplinary Research Facility (CIDRF), Sri Balaji Vidyapeeth Medical University, Mahatma Gandhi Medical College and Research Institute Campus, Puducherry, India

**Keywords:** Intraschisis fluid, X-linked retinoschisis, Proteome, Pathway analysis, Immune response, Inflammation

## Abstract

**Background:**

X-linked retinoschisis (XLRS) is a vitreoretinal degenerative disorder causing vision deterioration, due to structural defects in retina. The hallmark of this disease includes radial streaks arising from the fovea and splitting of inner retinal layers (schisis). Although these retinal changes are attributed to mutations in the retinoschisin gene, schisis is also observed in patients who do not carry mutations. In addition, the origin of intraschisis fluid, the triggering point of schisis formation and its progression are largely unknown still. So far, there is no report on the complete proteomic analysis of this fluid. Schisis fluid proteome could reflect biochemical changes in the disease condition, helping in better understanding and management of retinoschisis. Therefore it was of interest to investigate the intraschisis fluid proteome using high-resolution mass spectrometry.

**Methods:**

Two male XLRS patients (aged 4 and 40 years) underwent clinical and genetic evaluation followed by surgical extraction of intraschisis fluids. The two fluid samples were resolved on a SDS-PAGE and the processed peptides were analyzed by Q-Exactive plus hybrid quadrupole-Orbitrap mass spectrometry. Functional annotation of the identified proteins was performed using Ingenuity pathway analysis software.

**Results:**

Mass spectrometry analysis detected 770 nonredundant proteins in the intraschisis fluid. Retinol dehydrogenase 14 was found to be abundant in the schisis fluid. Gene ontology based analysis indicated that 19% of the intraschisis fluid proteins were localized to the extracellular matrix and 15% of the proteins were involved in signal transduction. Functional annotation identified three primary canonical pathways to be associated with the schisis fluid proteome viz., LXR/RXR activation, complement system and acute phase response signalling, which are involved in immune and inflammatory responses. Collectively, our results show that intraschisis fluid comprises specific inflammatory proteins which highly reflect the disease environment.

**Conclusion:**

Based on our study, it is suggested that inflammation might play a key role in the pathogenesis of XLRS. To our knowledge, this is the first report describing the complete proteome of intraschisis fluid, which could serve as a template for future research and facilitate the development of therapeutic modalities for XLRS.

**Electronic supplementary material:**

The online version of this article (doi:10.1186/s12014-017-9148-y) contains supplementary material, which is available to authorized users.

## Background

X-linked retinoschisis (XLRS) is a vitreoretinal disorder causing visual deterioration in the affected individuals, characterized by spoke-wheel pattern of the retina, splitting (schisis) within the retinal layers and reduced b-wave amplitude on Electroretinogram (ERG). It is a monogenic recessive disorder, predominantly affecting males. During the course of the disease, secondary complications like retinal detachment and vitreous hemorrhage may occur [1]. *RS1* (Retinoschisin1) is the gene implicated in XLRS and mutations in this gene have accounted for retinoschisis in most cases [2]. Functional studies have revealed that the gene product retinoschisin (RS1) is a cell–cell adhesion protein which likely helps in maintaining the structural organization of retina [3, 4]. Majority of the mutations in *RS1* gene produces an aberrant retinoschisin protein, which fails to perform its function [5]. However, there are few patients who do not harbour any mutation in the *RS1* gene, yet present severe clinical features of retinoschisis; bringing about the necessity to explore the disease mechanism [6]. Besides, disease severity greatly varies from one individual to the other irrespective of the type of *RS1* mutation or age [7]. Hence, there remains an ambiguity on the cue that triggers the formation of schisis cavity, its further progression and the accumulation of fluid into them.

Over the past few years, significant progress has been made in understanding the pathogenesis of XLRS in various aspects. Though extensive in vitro research at the molecular level has provided us profound insight on the expression and secretion of mutant retinoschisin, the actual proteomic changes in the affected eye has not been examined in detail [5, 8]. One such approach would be the analysis of intraschisis cavity fluid collected from XLRS patient. But, only little is known about the proteomic component of the intraschisis cavity fluid due to paucity of surgical samples. To date, there are only two reports on the characterization of this schisis fluid, wherein they have identified elevated levels of only two proteins, Cystatin C and Tenasin C owing to the limitations of the study methodology. One of the interesting findings is that these two proteins were detected in male (with *RS1* mutation) as well as female (without *RS1* mutation) retinoschisis patients; indicating that mutated RS1 might not be the sole reason for the intraretinal structural changes, but also due to some unknown mechanism which is yet to be explored [6, 9]. As compositional analysis of the schisis fluid is critical to understand the biochemical changes in the diseased eye, a comprehensive proteomic profiling is necessary to get a clue on the catalogue of putative biomarkers expressed and this could improve our knowledge on disease pathology as well as aid in developing therapeutic measures.

In our study, we have employed high resolution mass spectrometry to analyze the schisis cavity fluid of two male XLRS patients. We detected 770 nonredundant proteins in the schisis fluid with peptide as well as protein false discovery rates of 1%. Notably, Retinol dehydrogenase 14 (RDH14) was highly expressed in the intraschisis fluid. Subsequent functional annotation of these proteins using Ingenuity software has identified three major canonical pathways; LXR/RXR activation, complement system and acute phase response signalling, indicating the involvement of inflammation and active wound healing processes at the site of schisis. This data might serve as a valuable source of knowledge for future studies that focus on the pathophysiology of retinoschisis.

## Methods

### Clinical evaluation and surgical procedure

The two male patients underwent comprehensive eye examination such as detailed history, visual acuity, objective refraction, fundus examination using indirect ophthalmoscopy, Cirrus high definition-optical coherence tomography (OCT) (Carl-Zeiss Meditec AG, Jena, Germany) using 5-line raster scan (4096 A-scans) protocol and full field electroretinogram using Ganzfeld simulator. ERG was performed following the International Society for Clinical Electrophysiology of Vision guidelines [10]. Burian-allen contact lens electrodes were used to record the Dark-adapted 0.01 ERG, Dark-adapted 3.0 ERG, Dark-adapted 3.0 oscillatory potentials, Light-adapted 3.0 ERG and Light-adapted 3.0 flicker measurements.

**Fig. 1:**
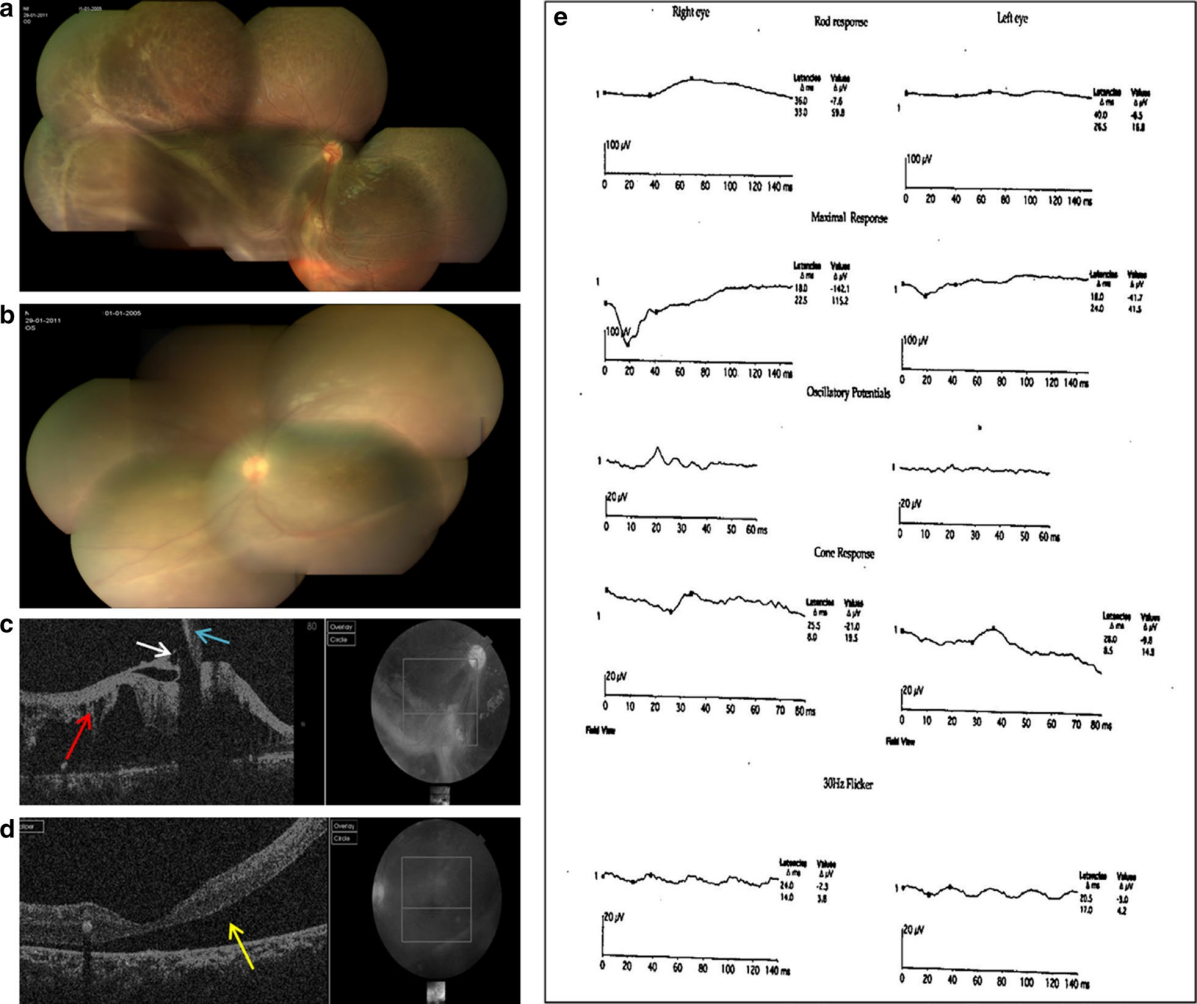
Preoperative fundus pictures, optical coherence tomography (OCT) images and electroretinogram (ERG) readings of patients I. **a** Montage fundus pictures of the right eye showing large schitic cavity inferotemporally with cystoid spaces seen superotemporally and temporally sparing the posterior pole. *Note* the inferior arcade being dragged down. **b** Montage fundus pictures of the left eye showing shallow retinal detachment involving the macula with bullous schisis seen inferotemporally and inferiorly. **c** OCT image of the right eye (*horizontal scan*) near the lower arcade showing preretinal traction (*blue arrow*) due to fibrosis and grossly distorted retina with large schitic spaces. *Note* strands of retinal tissues bridging between the inner retina and thinned outer retinal layer adjacent to retinal pigment epithelium (*red arrow*). A small epiretinal membrane is seen in front of the retina (*white arrow*). **d** *Horizontal* OCT image of the left eye showing full thickness and retinal detachment involving the fovea (*yellow arrow*). Note the lack of strands extending between the detached retina and retinal pigment epithelium unlike in schisis. **e** ERG of both eyes showing classical negative waveform due to grossly reduced or absent b-wave amplitude

Vitreoretinal surgery was planned for both the male patients to collect sequelae of retinoschisis. Informed consent for surgery and peripheral blood sample collection was obtained either from the patient or family member. The entire study protocol was approved by the institutional review board (Vision Research Foundation, Chennai, India) as well as the ethics committee (Ref No. 202-2009-P) and adhered to the tenets of declaration of Helsinki. The details of the surgery and schisis fluid collection are as follows:

Patient I (aged 4 years), presented with bilateral retinoschisis. He had poor vision since early childhood; his best corrected visual acuity was 6/38 in the right eye and 1/60 in the left eye. He had a refractive error of +5.5DS and +7.5DS in the right eye and left eye respectively. In the left eye there was a large cyst overhanging the macula, while in the right eye the macula was attached. Hence surgery was planned in the left eye externally. There was no retinal detachment in this case. After the sclerotomies were made for vitreoretinal surgery, a needle was passed transclerally into the intraschitic space and the fluid was aspirated under direct visualization using a 2 ml syringe until the schisis cavity collapsed. Throughout this procedure, care was taken not to suck the vitreous gel and the infusion was shut off to avoid dilution. Once the needle was withdrawn the intraocular pressure was restored with balanced salt solution infusion and surgery proceeded with. In this case, there was no chance of contamination with vitreous or subretinal fluid as the needle was passed transclerally into the intraschisis cavity. Representative fundus pictures, OCT images and ERG readings of patient I are shown in Fig. 1.

**Fig. 2:**
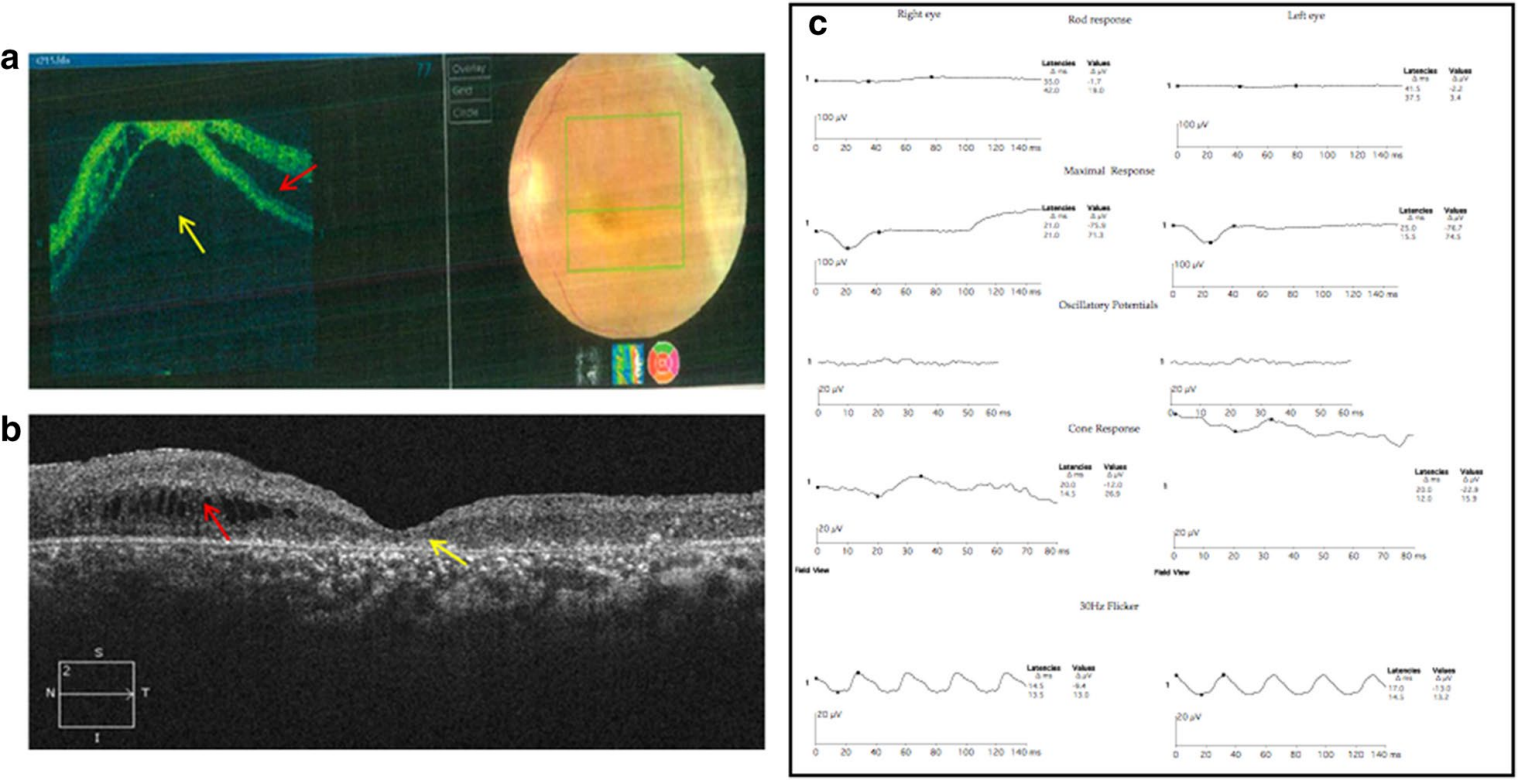
Representative optical coherence tomography (OCT) images and electroretinogram (ERG) readings of patient II. **a** Preoperative OCT image of the *left eye* with corresponding fundus picture, suggestive of detached macula (*yellow arrow*) and schisis (*red arrow*). **b** Postoperative OCT image of the *left eye* showing attached macula (*yellow arrow*) and partly collapsed schisis (*red arrow*). **c** ERG of the right and *left eye* showing grossly delayed and reduced single flash rod responses and negative waveform in combined responses

Patient II (aged 40 years), had bilateral retinoschisis. He had diminution of vision in both the eyes since the age of 7. On a recent examination, his best corrected visual acuity was 3/60 with a refractive error of +9.00DS/−1.25DC*90 and +4.50DS/−2.00DC*90 in the right eye and left eye respectively. The right eye had large inner layer breaks, but no retinal detachment. The left eye developed rhegmatogenous retinal detachment due to outer retinal break and hence was subjected to surgery. After the sclerotomies are made for vitreoretinal surgery, attempt was made to collect uncontaminated schitic fluid. A needle connected to a syringe was passed across the vitreous cavity into the schitic cavity through a pre-existing inner retinal layer break or by penetrating the inner layers if no breaks existed. Once the needle tip was positioned within the schitic cavity, gentle aspiration of the schisis fluid was done under direct visualization using a 2 ml syringe until the schitic cavity collapsed. Throughout this procedure, care was taken not to suck the vitreous gel and the infusion was shut off to avoid dilution. Once the needle was withdrawn the intraocular pressure was restored with balanced salt solution infusion and surgery proceeded with. Representative OCT images and ERG readings of patient II are shown in Fig. 2.

Contamination of the schisis fluid with blood during surgical procedure was excluded by subjecting the samples to automated complete blood count hematology analyzer which is based on the Coulter VCS (volume, conductivity and scatter) technology (LH 750, Beckman Coulter, Fullerton, CA, USA).

### Genetic screening

Genomic DNA was extracted from peripheral blood samples of both the patients using Nucleospin kit (Macherey–Nagel, Duren, Germany) according to the manufacturer’s instruction. Primers sequences for all the exons of *RS1* gene and their respective PCR cycling profiles were obtained from the literature [11]. The PCR products were then bidirectionally sequenced using a cycle sequencing kit (Big Dye Terminator v3.0 Ready, Applied Biosystems, Foster City, CA, USA) and an ABI PRISM 3100 *Avant* genetic analyzer (Applied Biosystems Inc.). The output was compared with *RS1* reference sequence from the Ensembl database.

### Sample digestion and processing for mass spectrometry

**Fig. 3:**
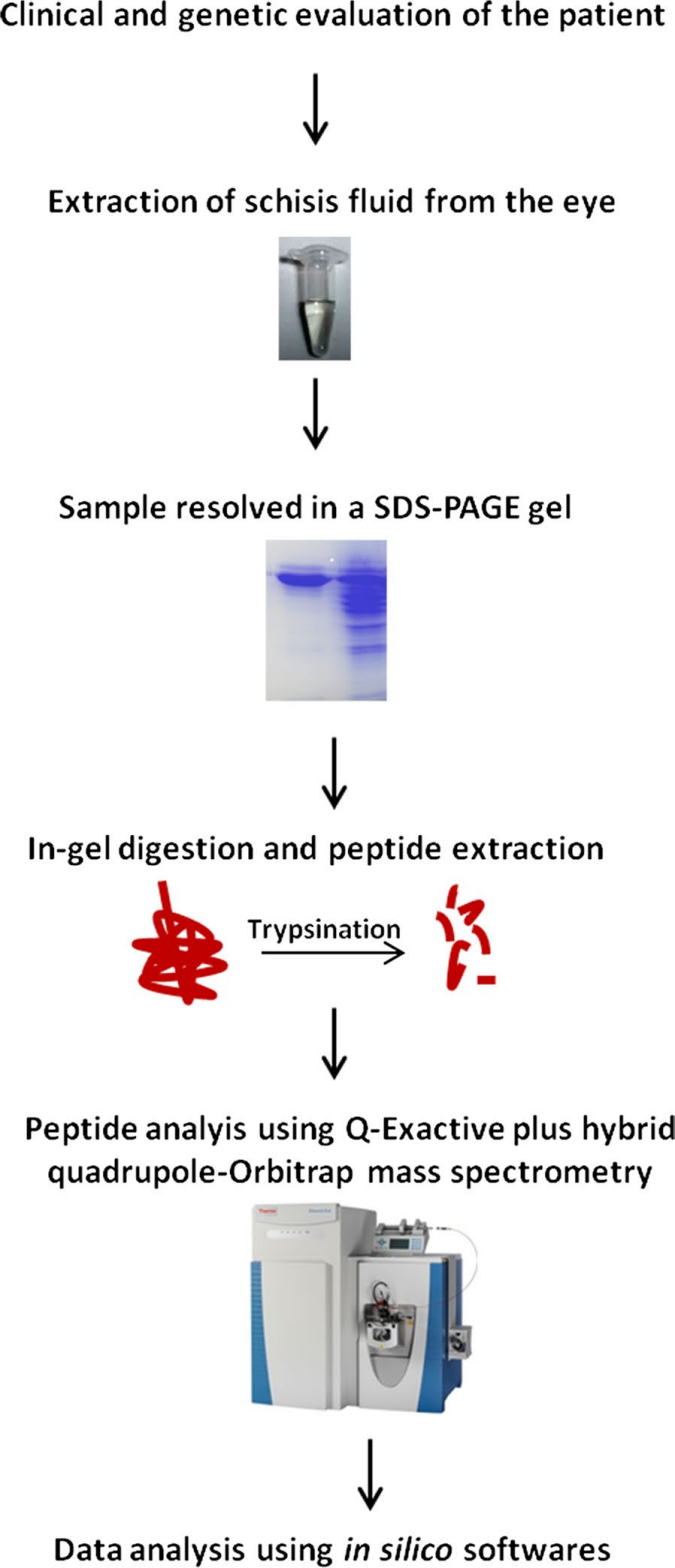
Experimental design of the proteomic characterization and analysis of intraschisis fluid

Two schisis fluid samples obtained from each of the patient by surgical intervention were immediately stored at −80 °C. The protein concentration of the fluid samples was estimated by Bradford assay kit (Sigma-Aldrich, St. Louis, MO, USA) and they were further processed individually for mass spectrometry protocol, followed by data analysis as illustrated in Fig. 3.

50 µg of each intraschisis fluid sample was separated on a 4–12% gradient SDS-PAGE gel (NuPAGE, Life science technologies, Carlsbad, CA, USA), followed by coomassie staining. Each lane was cut into 22 pieces and in-gel digested with trypsin according to Shevchenko and colleagues [12]. The proteins in each gel piece were reduced with 10 mM dithiothreitol (Sigma-Aldrich Inc.) for 50 min at 56 °C, alkylated with 55 mM iodoacetamide (Sigma-Aldrich Inc.) for 20 min at room temperature and digested with 10 mM trypsin for 45 min at 4 °C. Following this, the gel pieces were immersed in ammonium bicarbonate (Sigma-Aldrich Inc.) and incubated overnight at 37 °C. Afterwards, the peptides from each gel piece were extracted using 80% acetonitrile and 0.5% formic acid (Sigma-Aldrich Inc.). The extracted peptides were vacuum dried and then dissolved in 20 µl of the loading buffer (5% acetonitrile and 0.1% formic acid). Likewise, the schisis fluids were also processed by in-sol digestion procedure, where the sample was acetone precipitated and then dissolved in 1% Rapigest SF (Waters, Milford, Massachusetts, USA) at 60 °C for 15 min. Following this, the sample was reduced, alkylated and trypsin digested. After arresting the activity of trypsin using formic acid, the sample was vacuum concentrated and then resuspended in the loading buffer as described earlier.

### LC-MS/MS analysis

The peptides were analyzed on Q-Exactive plus hybrid quadrupole-Orbitrap mass spectrometer (Thermo Fisher Scientific, Germany) coupled with a Nano-LC pump (EASY-nLC). The peptides were pre-concentrated on a trap column (0.15 mm ID  ×  20 mm self-packed with Reprosil-Pur120 C18-AQ 5 μm, Dr. Maisch GmbH, Ammerbuch-Entringen, Germany) at 10 µl/min in loading buffer and then separated by an analytical column (0.075 mm ID  ×  300 mm self-packed with Reprosil-Pur 120 C18-AQ, 1.9 μm, Dr. Maisch GmbH) using a linear gradient from 5 to 44% buffer (95% acetonitrile and 0.1% formic acid) at flow rate of 320 nl/min. The mass spectrometer was operated in data-dependent acquisition mode. Full scan MS spectra were acquired in the range of 350–1600 m/z at a resolution of 70,000. The top 15 (in-sol digested samples) and top 20 (in-gel digested samples) most intense peaks from the survey scan were selected for fragmentation with Higher-energy Collisional Dissociation with the normalized collision energy of 28% and isolation window 1.6 m/z. Dynamic exclusion time for precursor ions selected for MS/MS fragmentation was 30 s. Automatic gain control target value and maximum ion injection time for MS and MS/MS were 1 × 10^6^ and 1 × 10^4^ respectively.

### Data analysis

Proteome Discoverer software suite 1.3 (Thermo Scientific, Bremen, GmBH) was used for protein identification through combined Mascot and Sequest search algorithms against Human RefSeq 70 protein database with added contaminants. The criteria used for search in the Mascot algorithm are as follows: Trypsin was used as protease with maximum 1 missed cleavage allowed. Protein N-terminal acetylation, deamidation of asparagine and glutamine, and Oxidation of methionine were fixed as dynamic modifications whereas carbamidomethyl of Cysteine was kept as static modification. The protein list was further filtered by applying protein level false discovery rate cut-off of 1%.

### Bioinformatics analysis

Gene Ontology (GO) study of the proteome was performed using the functional enrichment and network analysis tool, FunRich (Functional Enrichment Analysis Tool) [13]. Normalised spectral abundance factor (NSAF) was calculated and the proteins were arranged according to the NSAF values. In general, large proteins usually generate more peptides and therefore more spectral counts than smaller proteins. So, the number of spectral counts for each protein needs to be divided by the mass or protein length, which defines the spectral abundance factor (SAF). However, to account for the variability between runs, individual SAF values was normalized to one by dividing by the sum of all SAFs for proteins in the sample, which gives the NSAF value. The NSAF value was calculated as follows:

$$ \left( {\text{NSAF}} \right)k = \frac{{\left( {\frac{SpC}{L}} \right)k}}{{\mathop \sum \nolimits_{i = 1}^{N} \left( {\frac{SpC}{L}} \right)i}} $$

where the total number of MS spectra matching peptides from protein *k* (*SpC*), was divided by the protein’s length (L), then divided by the sum of SpC/L for all N proteins [14]. The pathway analysis was generated through the use of QIAGEN’s Ingenuity Pathway Analysis (IPA, QIAGEN Redwood City, CA, USA) to identify the canonical pathways associated with the dataset [15].

## Results

### Genotype evaluation

*RS1* gene screening of the coding regions revealed a novel mutation, p. D126H in patient I and a reported mutation, p.R197H in patient II. These two mutations were found to be located in the discoidin domain of RS1, which might interfere with the secretion and function of the protein.

### Schisis fluid proteome analysis

The schisis fluids collected from the two XLRS patients were individually processed and analyzed by high resolution mass spectrometry. It was not possible to obtain an appropriate control for comparison, as this is a pathological fluid present only in the diseased condition. Complete proteomic profiling of the schisis fluid detected an extensive list of 770 non-redundant proteins, given in the Additional file 1: Table S1. The proteins were arranged according to their normalised spectral abundance factor (NSAF) value, which is a label-free method for effective protein quantification. A high NSAF value indicates abundant expression of a particular protein in the sample [14]. Representative list of proteins with high NSAF value and their clinical relevance is given in Table 1.

**Table 1:** Representative list of proteins detected in the intraschisis fluid

Sl. no.	Protein symbol	Description	Clinical relevance
1	RDH14	Retinol dehydrogenase 14 [Homo sapiens]	Visual cycle [16]
2	SGCE	Epsilon-sarcoglycan isoform 2 [Homo sapiens]	Myoclonus dystonia [46]
3	STK26	Serine/threonine-protein kinase 26 isoform 2 [Homo sapiens]	–
4	TENM1	Teneurin-1 isoform 3 [Homo sapiens]	–
5	ALMS1	Alstrom syndrome protein 1 [Homo sapiens]	Alstorm syndrome [47, 48]
6	ZFP90	Zinc finger protein 90 homolog isoform 1 [Homo sapiens]	–
7	GRIN1	Glutamate receptor ionotropic, NMDA 1 isoform GluN1-4a precursor [Homo sapiens]	Intellectual disability [49]
8	QSER1	Glutamine and serine-rich protein 1 [Homo sapiens]	–
9	ESCO1	N-acetyltransferase ESCO1 [Homo sapiens]	Bladder cancer [50] Endometrial cancer [51]
10	KIF4A	Chromosome-associated kinesin KIF4A [Homo sapiens]	–
11	MSC	Musculin [Homo sapiens]	–
12	TBC1D32	Protein broad-minded [Homo sapiens]	–
13	SIGLEC7	Sialic acid-binding Ig-like lectin 7 isoform 2 precursor [Homo sapiens]	–
14	PRICKLE4	Prickle-like protein 4 [Homo sapiens]	Progressive myoclonus epilepsy-ataxia syndrome [52]
15	INSR	Insulin receptor isoform Short preproprotein [Homo sapiens]	Familial hyperinsulinemic hypoglycaemia [53] Rabson-Mendenhall syndrome [54] Leprechaunism [55] Non-insulin-dependent diabetes mellitus [56] Insulin-resistant diabetes mellitus with acanthosis nigricans [57]
16	HEPACAM	Hepatocyte cell adhesion molecule precursor [Homo sapiens]	Megalencephalic leukoencephalopathy with subcortal cysts [58]
17	KIAA1147	Protein LCHN [Homo sapiens]	–
18	CAMSAP3	Calmodulin-regulated spectrin-associated protein 3 isoform 1 [Homo sapiens]	–
19	CAPN1	Calpain-1 catalytic subunit [Homo sapiens]	–
20	ANKRD24	Ankyrin repeat domain-containing protein 24 [Homo sapiens]	–

### Gene ontology analysis

**Fig. 4:**
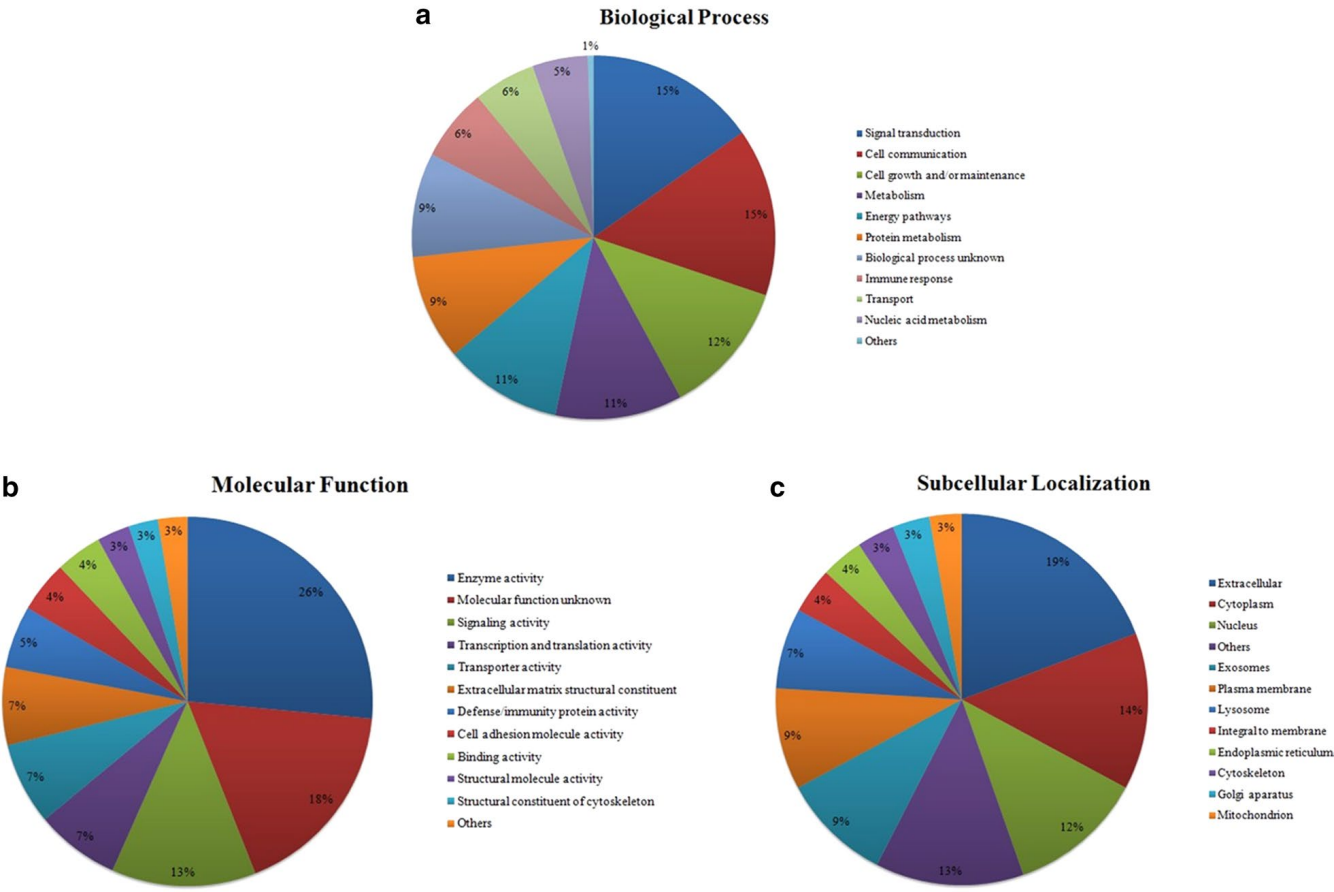
Gene ontology based classification of proteins identified in the intraschisis fluid. **a** Biological process, **b** molecular function and **c** subcellular localization

Gene Ontology based analysis of the intraschisis fluid proteome was performed using FunRich to segregate proteins on the context of subcellular localization, molecular class and biological processes. On the basis of subcellular localization, it was observed that a majority of the proteins localized to the extracellular matrix (19%), cytoplasm (14%) and the nucleus (12%). When categorized based on molecular function, about 26% of them were known to have enzymatic activity while 13% had signaling activity. Most of the other proteins belonged to the class of transporter activity, cell adhesion, defense or immunity protein, transcription and translation activity, etc. Classification based on biological process showed that 15% of the proteins were involved in signal transduction, 15% in cell communication and 12% in cell growth or maintenance. The distribution of proteins under each category is represented in Fig. 4.

### Functional annotation

In order to obtain a functional overview of the complete intraschisis fluid proteome, we employed Ingenuity pathway analysis software. Table 2 shows the disease conditions associated with the proteins found in the schisis fluid and Table 3 shows their molecular and cellular functions. Core analysis identified 5 pathways, based on the percentage of individual proteins associated with the respective pathway (Table 4). The most significant canonical pathways associated with the schisis fluid proteome were LXR/RXR activation (p = 4.41E−28, 42 proteins), complement system (p = 1.11E−23, 23 proteins) and acute phase response signalling (p = 1.43E−27, 48 proteins). Candidate proteins identified in the retinoschisis fluid which are involved in the abovementioned pathways are shown as pictorial representations in Additional files 2, 3 and 4: Figure S1, Figure S2 and Figure S3.

**Table 2:** Diseases and disorders related to the proteins detected in the intraschisis fluid

Name	Number of molecules detected
Neurological disease	264
Hereditary disease	245
Metabolic disorder	181
Psychological disorder	178
Developmental disorder	127

**Table 3:** Molecular and cellular functions related to the proteins detected in the intraschisis fluid

Names	Number of molecules detected
Cellular growth and proliferation	216
Cell death and survival	208
Cellular movement	161
Cellular development	119
Cellular function and maintenance	117

**Table 4:** Candidate proteins involved in the predominant canonical pathways that were identified in the intraschisis fluid

Pathway	p value	Number of candidate proteins	Protein symbols of candidate proteins detected in RS fluid
LXR/RXR activation	4.41E − 28	42/121	A1BG, AGT, AHSG, ALB, AMBP, APOA1, APOA2, APOA4, APOB, APOC3, APOD, APOE, APOH, APOL1, APOM, C3, C9, C4A/C4B, CD14, CLU, FASN, FGA, GC, HPX, ITIH4, KNG1, LBP, LDLR, LYZ, ORM1, ORM2, PLTP, PON1, RBP4, S100A8, SAA4, SERPINA1, SERPINF1, SERPINF2, TF, TTR, VTN
Acute phase response signalling	1.43E − 27	48/168	A2M, AGT, AHSG, ALB, AMBP, APOA1, APOA2, APOH, C2, C3, C5, C9, C1R, C1S, C4A/C4B, CFB, CP, CRABP1, F2, FGA, FGB, FGG, FN1, FTL, HP, HPX, HRG, ITIH2, ITIH3, ITIH4, KLKB1, LBP, ORM1, ORM2, PLG, RBP1, RBP3, RBP4, SAA4, SERPINA1, SERPINA3, SERPIND1, SERPINF1, SERPINF2, SERPING1, SOD2, TF, TTR
Complement system	1.11E − 23	23/36	C2, C3, C5, C6, C7, C9, C1QA, C1QB, C1QC, C1R, C1S, C4A/C4B, C8A, C8B, C8G, CD59, CFB, CFD, CFH, CFI, ITGAM, MASP2, SERPING1

## Discussion

### High-abundance proteins in the schisis fluid

Among the list of upregulated proteins in the schisis fluid, RDH14 had the highest NSAF value of 23.51. RDH14 belongs to dual-specificity retinol dehydrogenases that catalyze the conversion of all-trans- and cis-retinol into retinal and regulate the production of retinoic acid as well. This reaction is the rate limiting step of the visual cycle [16]. Although variations in *RDH14* are benign and not disease causing, mutations in *RDH5* and *RDH12* are known to be associated with fundus albipunctatus and leber congenital amaurosis [17, 18].

Few other significant proteins with high NSAF value include PTPRS (Receptor-type tyrosine-protein phosphatase Sigma isoform 3 precursor; NSAF value—8.95), CRYGC (Gamma-crystallin C; NSAF value—6.11), APOB (Apolipoprotein B-100 precursor; NSAF value—3.55) and F5 (Coagulation factor V precursor; NSAF value—2.91). However retinoschisin, a major secretory protein of the retina was not detected in both the schisis fluid samples. It is reported that certain *RS1* mutations affect the secretory phenomenon of retinoschisin, resulting in complete intracellular retention of RS1 [5]. Considering these facts, the protein expression profile of these two mutations was investigated by creating mutant constructs which were then transfected into COS7 cells. The expression of the mutant and wild type constructs were studied by analyzing the intracellular and secretory fractions using immunoblotting. Both the RS1 mutants were detected only in the intracellular fraction, while, the wild type protein was detected in the intracellular as well as in the secretory fraction. (Unpublished observation; Sudha D and Jayamuruga Pandian A). However, owing to the tissue damage or injury occurring during the disease progression, there is a possibility that the intracellularly retained mutant RS1 might percolate into the schisis cavity fluid. Nevertheless, RS1 wasn’t detected in the mass spectrometric analysis maybe due to insignificant amount of the disseminated intracellular RS1 in the intraschisis fluid collected from these patients.

### Schisis fluid proteome and the vitreous proteome

The actual source of the schisis fluid that gets accumulated in XLRS condition is still not understood. It is presumed that the accumulation of fluid inside the schisis cavity might be caused by the infiltration of vitreous fluid due to the loss of retinal membrane integrity in XLRS condition. If so, the schisis fluid collected from XLRS patients would have proteins expressed in the vitreous fluid as well as specific proteins expressed exclusively during the disease condition. Moreover, there is no possibility of obtaining a disease matched control sample with which the schisis proteome could be compared. Hence, it was of interest to identify and distinguish the candidate proteins specifically expressed in XLRS. Due to practical difficulties in obtaining vitreous sample from the same patient, we compared the schisis proteome with already published vitreous proteome, though the methodology and proteomic techniques varied between the studies. For this purpose, a consolidated data of the vitreous proteome (2854 proteins) was created based on published literature, which served as the template for comparison [19–22]. To further improve the stringency, only those proteins (725) that were identified in at least two independent research works were considered for the comparative analysis. Out of 770 proteins in the intraschisis fluid, 352 (46%) were common to the vitreous as well as schisis fluid proteome, while 416 proteins were found only in the schisis fluid proteome (Additional file 5: Figure S4). This might indicate that the intraschisis fluid could be a part of the vitreous which has seeped into the schisis cavity. Nonetheless, this observation needs to be substantiated by further experiments. Further, we analyzed the schisis fluid-specific proteins with the proteome data sets of various ocular tissues such as the retina, ciliary body, iris, retinal pigment epithelium, choroid, sclera and optic nerve [23–25]. On comparison, we found that 257 (33%) schisis fluid proteins were not detected in any of the ocular tissues including the vitreous fluid.

Attempts to characterize the schisis fluid has been earlier carried out by Drenser and colleagues using SDS-PAGE and HPLC analysis, wherein two unique proteins were identified viz., Cystatin C and Tenasin C. Cystatin C is a cysteine protease inhibitor activated during infection and inflammation, while Tenasin C is an extracellular matrix glycoprotein, induced during tumorgenesis, inflammation or infection [6, 9]. Our study identified Cystatin C and Teneurin-1, which belongs to the Tenascin family. The finding of both these proteins in the schisis fluid is consistent with the previous reports, further supporting the view that these proteins are upregulated during tissue damage and inflammation, the underlying pathology in XLRS.

### Signalling pathways

Currently, our knowledge of the processes by which the schisis is initially triggered is very poorly understood. Therefore, the first step towards the development of an effective therapeutic agent would be determining the underlying disease mechanisms in order to identify the most appropriate means for intervention. Our functional annotation analysis of the schisis fluid proteome identified three major pathways—LXR/RXR activation, complement system and acute phase response signalling.

The retinoid X receptors (RXRs) and liver X receptors (LXRs) are nuclear receptors that regulate retinoic acid-mediated gene activation [26]. Both LXR/RXR and the FXR/RXR pathways (farnesoid X receptor) are involved in lipid metabolism, inflammation and cholesterol conversion to bile acid. Cholesterol plays an important role in CNS synaptogenesis and is essential for optimal neurotransmitter release. Defective cholesterol homeostasis in brain is associated with neurodegeneration leading to disorders like Alzheimer’s disease [27]. LXR knockout mouse has been shown to develop neurodegenerative changes. LXR/RXR activation has also been observed in age-related macular degeneration (AMD) cases [28]. AMD and XLRS share a number of probable disease related proteins such as CFH, HTRA1, C2, CFB, APOE, etc. [29, 30]. Likewise upregulation of genes involved in LXR/RXR activation pathway has been observed in a mouse model of glaucoma exhibiting ganglion cell death, increased intraocular pressure and pigment dispersion [31].

The complement system represents a major component of immunity, playing a vital role in the defence against infection and in the modulation of immune and inflammatory responses. In addition to its well-established functions, the complement system has been recently implicated in a variety of pathophysiological processes like ischemia, sepsis, stroke, autoimmunity and inhibition of neovascularisation [32]. Within the ocular microenvironment, the alternative complement cascade is under a continuous low-level state of activation which allows for this pathway to have crucial immune surveillance without causing any damage to self-tissue. However, the expression of complement regulatory proteins causes the increased deposition of C3 (complement component 3) and activation of the membrane attack complex thereby leading to diseased conditions like corneal inflammation, AMD or diabetic retinopathy [33–35]. Besides, complement also stimulates TGFβ which is the most important ligand in fibrotic diseases of the eye, which in some cases causes retinal detachment, where the photoreceptors undergo apoptosis and programmed necrosis. Control of complement activation at the level of C3 convertase has been proven to be sufficient in preventing complement mediated intraocular inflammation [36]. It is noteworthy to mention that retinoschisin knock out mouse retina displayed upregulation of C1qb and MAP Erk1/2 kinases indicating the involvement of complement activation in retinoschisis [37].

The acute phase response is a rapid inflammatory response that provides protection against infection, tissue injury, neoplastic growth or immunological disorders. Specific role of this pathway in ocular conditions such as Behcet disease, diabetic retinopathy and retinal detachment have been established, where the early stress-response genes and specific signalling pathways are known to be activated [38, 39]. This adaptive response may enable the photoreceptor cells to survive the acute phase of a retinal detachment, and it is the breakdown of these protective mechanisms that leads to the ultimate death of the cell [40].

### Pathophysiology of XLRS: hypothetical mechanism

RDH14 shares ~40–46% sequence identity with RDH11 and RDH12, which have retinol dehydrogenase activity. As RDH14 is highly expressed in other human tissues, it is considered to be more essential in maintaining the retinoid homeostasis than RDH11. Hence an upregulation of RDH14 (detected in the schisis fluid) might catalyze the excessive production of retinoic acid. Retinoic acid exerts its action by serving as an activating ligand of nuclear retinoic acid receptors such as RAR (retinoic acid receptor) and RXR, which mediate the retinoid signalling pathways [41]. RXR is capable of forming heterodimers with LXR, FXR etc. and therefore such ligand activation has pleiotrophic effects on numerous biological pathways. The LXR/RXR and FXR/RXR pathway activated by one of the agonists of either LXR or RXR or FXR regulates the transcription of genes such as APOE (apolipoprotein E), C3 etc. that might trigger the complement pathway [28, 42]. Evidence from the schisis fluid proteome indicates the possible involvement of both the classical or alternate complement pathway, which may cause tissue injury in the disease condition. As a result the stress response genes associated with the acute phase signalling is likely to be evoked as a protective mechanism. The presence of acute phase proteins in the system might consequently bring about the complement-mediated elimination of cell debris and aid in modulating the host’s immune response [43–45]. Thus the over expression of RDH14 in the schisis fluid is likely to play a role in the elicitation of these canonical pathways contributing to the disease pathology. Nevertheless, an extensive investigation of this hypothesis is indispensible to correlate the speculated mechanism to the disease pathology.

## Conclusion

Based on our analysis and research, it is proposed that the abovementioned highly abundant proteins in addition to those exclusively expressed in the schisis fluid could serve as clinical indicators of the disease. Of these, RDH14 might play a central role in the disease pathogenesis owing to its putative role in the visual cycle and regulation of retinoic acid production. However, a thorough investigation and characterization of these proteins and pathways are necessary to be implicated as potential biomarkers of the disorder.

A possible strategy to combat disease progression could be targeting the specific activation products of the pathways or their respective receptors by the help of antagonists. One such target would be the FXR, as it stimulates C3 of the complement pathway. Of particular interest are those antagonists that could act on the complement system as it has been shown to initiate cell death pathways in a number of disease models including acute lung injury, myocardial perfusion injury etc. On the contrary, agonists may also act as therapeutic drugs. For instance, oxysterol, an LXR agonist has shown to reduce inflammation through the regulation of macrophage activity.

Therefore, modulating and selectively blocking certain pathways using the relevant agonist or antagonist may aid in reducing inflammation at the disease site, and serve as a therapeutic drug to reduce the disease severity, if not its occurrence. Nevertheless, the critical challenge for developing effective and safe therapeutics is to balance the beneficial effects by inhibition of these pathways with the preservation of their necessary functional activity. Taken together, we suggest that immune response and inflammation might play a key role in the pathogenesis of XLRS and modulation of this might help in the management of the disease.

## Additional files

**Additional file 1: Table S1.** Complete list of proteins detected in the intraschisis fluid.

**Additional file 2: Figure S1.** Pictorial representation of LXR/RXR pathways, highlighting the candidate proteins detected in the intraschisis fluid.

**Additional file 3: Figure S2.** Pictorial depiction of the complement pathway, highlighting the candidate proteins detected in the intraschisis fluid.

**Additional file 4: Figure S3.** Pictorial illustration of the acute phase signalling, highlighting the candidate proteins detected in the intraschisis fluid.

**Additional file 5: Figure S4.** Venn diagram illustrating the number of proteins that overlaps between the vitreous and intraschisis fluid proteome.

## Abbreviations

XLRS: X-linked retinoschisis
ERG: electroretinogram
RS1: retinoschisin
RDH: retinol dehydrogenase
OCT: optical coherence tomography
DNA: deoxyribonucleic acid
PCR: polymerase chain reaction
VCS: volume, conductivity and scatter
SDS-PAGE: sodium dodecyl sulphate polyacrylamide gel electrophoresis
LC-MS/MS: liquid chromatography mass-spectrometry
DS: dioptres sphere
DC: dioptres cylinder
NSAF: normalized spectral abundance factor
SAF: spectral abundance factor
HPLC: high performance liquid chromatography
RXR: retinoid X receptor
LXR: liver X receptor
FXR: farnesoid X receptor
AMD: age-related macular degeneration
RAR: retinoic acid receptor
C3: complement component 3
APOE: apolipoprotein E
