# Supplementary figures and images for: Proteomic profiling of human intraschisis cavity fluid
Source: Clin Proteomics. 2017 Apr 24;14:13. doi: 10.1186/s12014-017-9148-y (PMC5404285; doi:10.1186/s12014-017-9148-y)

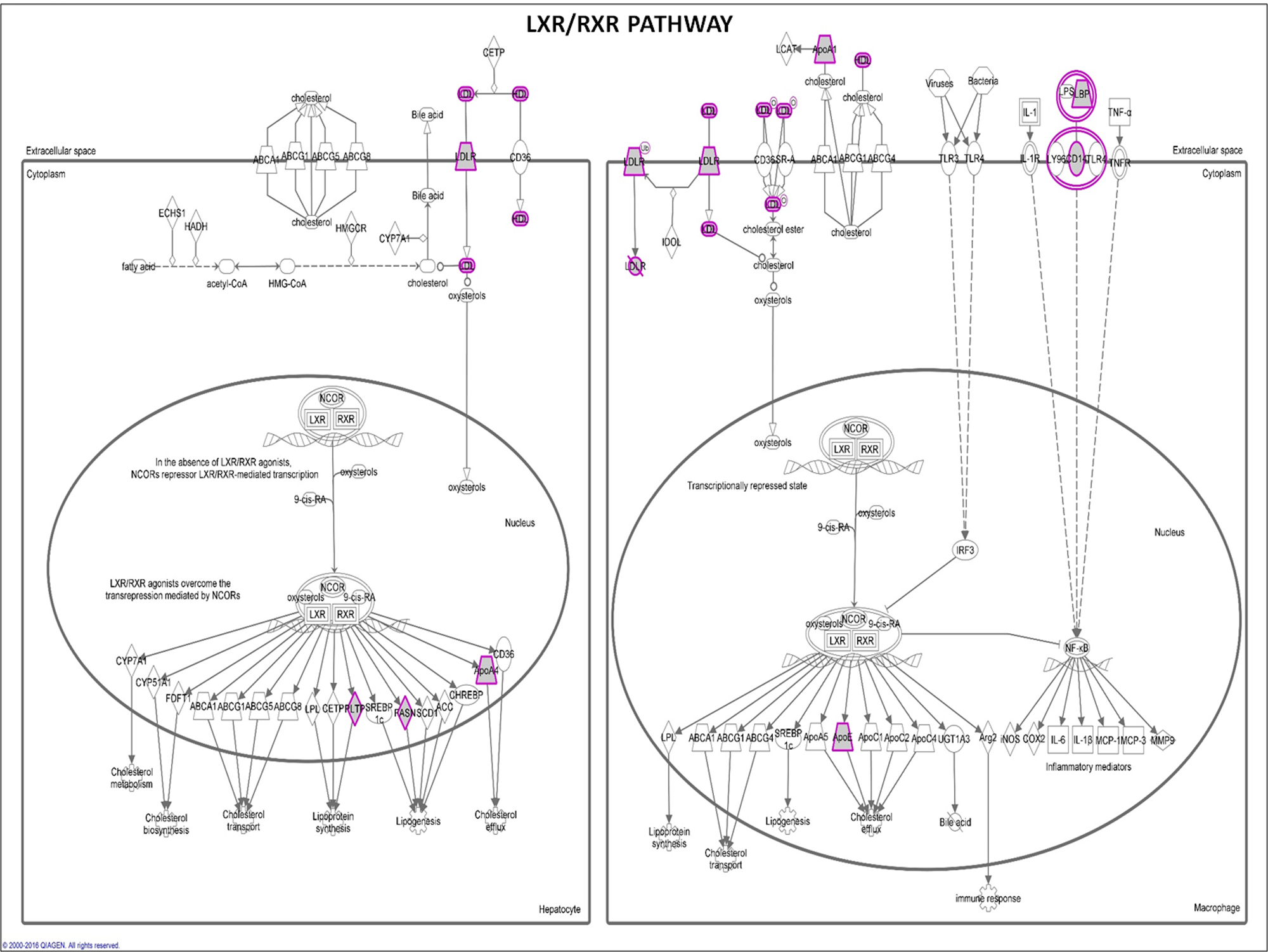

Supplement: Supplementary file 2 — Additional file 2: Figure S1. Pictorial representation of LXR/RXR pathways, highlighting the candidate proteins detected in the intraschisis fluid. [file 12014_2017_9148_MOESM2_ESM.tif]

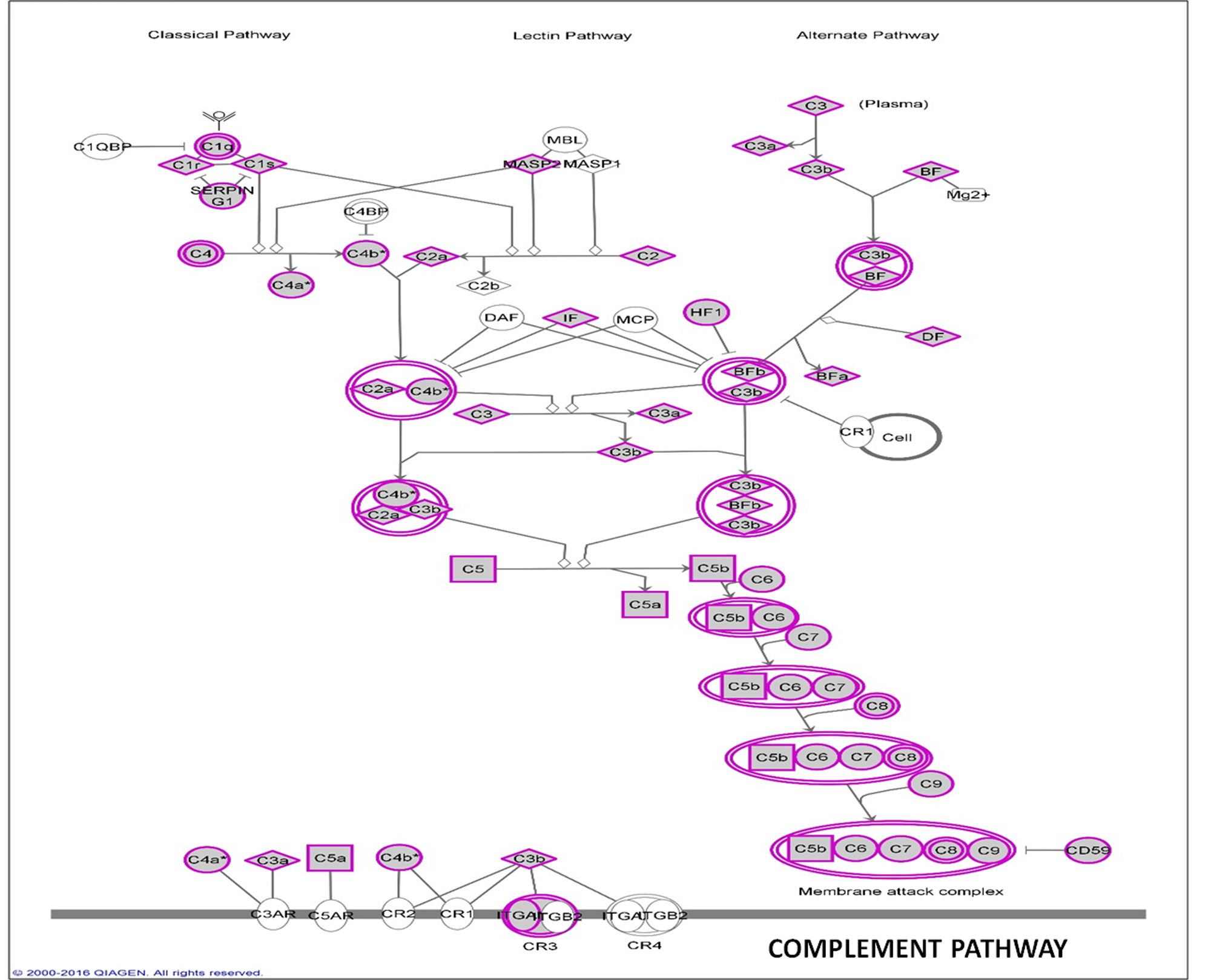

Supplement: Supplementary file 3 — Additional file 3: Figure S2. Pictorial depiction of the complement pathway, highlighting the candidate proteins detected in the intraschisis fluid. [file 12014_2017_9148_MOESM3_ESM.tif]

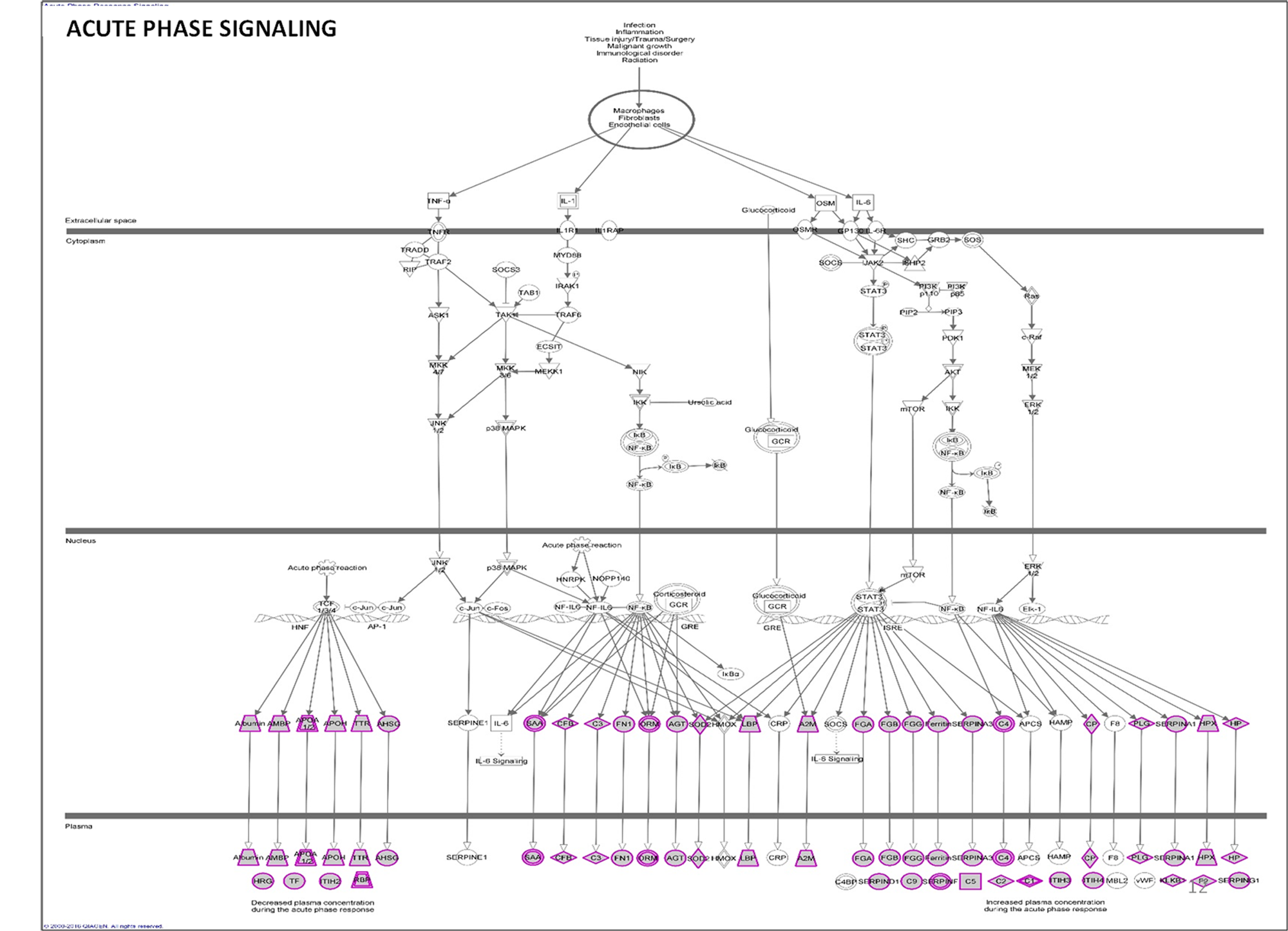

Supplement: Supplementary file 4 — Additional file 4: Figure S3. Pictorial illustration of the acute phase signalling, highlighting the candidate proteins detected in the intraschisis fluid. [file 12014_2017_9148_MOESM4_ESM.tif]

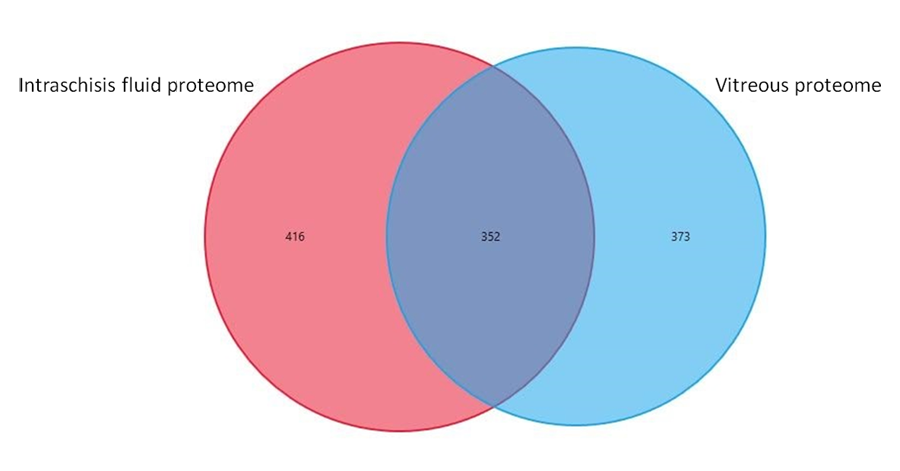

Supplement: Supplementary file 5 — Additional file 5: Figure S4. Venn diagram illustrating the number of proteins that overlaps between the vitreous and intraschisis fluid proteome. [file 12014_2017_9148_MOESM5_ESM.tif]
